# Effects of Eight Weeks of High Intensity Functional Training on Glucose Control and Body Composition among Overweight and Obese Adults

**DOI:** 10.3390/sports7020051

**Published:** 2019-02-22

**Authors:** Yuri Feito, Pratik Patel, Andrea Sal Redondo, Katie M. Heinrich

**Affiliations:** 1 Department of Exercise Science and Sport Management, Kennesaw State University, Kennesaw, GA 30144, USA; 2 New York Football Giants, East Rutherford, NJ 07073, USA; pratik.patel@giants.nfl.net; 3 Facultad de Ciencias de la Salud, Universidad Europea Miguel de Cervantes, Valladolid 47012, Spain, andreasal183@gmail.com; 4 Department of Kinesiology, Kansas State University, Manhattan, KS 66506, USA

**Keywords:** fitness, diabetes, CrossFit, exercise

## Abstract

High-intensity exercise has been found to positively influence glucose control, however, the effects of high-intensity functional training (HIFT) for overweight and obese sedentary adults without diabetes is unknown. The purpose of this study was to examine changes in body composition and glucose control from eight weeks of aerobic and resistance training (A-RT) compared to HIFT. Session time spent doing daily workouts was recorded for each group. Baseline and posttest measures included height, weight, waist circumference, dual X-ray absorptiometry (body fat percentage, fat mass, lean mass), and fasting blood glucose. Participants completing the intervention (78%, n = 9 per group) were 67% female, age = 26.8 ± 5.5 years, and had body mass index = 30.5 ± 2.9 kg/m^2^. Fasting blood glucose and 2-h oral glucose tolerance tests were used as primary outcome variables. On average, the HIFT group spent significantly less time completing workouts per day and week (ps < 0.001). No significant differences were found for body composition or glucose variables within- or between-groups. Even though our findings did not provide significant differences between groups, future research may utilize the effect sizes from our study to conduct fully-powered trials comparing HIFT with other more traditional training modalities.

## 1. Introduction

As health care costs attributable to diabetes continue to rise [1,2], it is imperative to examine new approaches to prevent this chronic disease. Evidence from the Diabetes Prevention Program (DPP) demonstrates that a lifestyle intervention significantly reduces the cumulative incidence of diabetes close to 60%, in comparison to 30% in a group using medication (Metformin) alone [3]. Moreover, the DPP study also demonstrated that lifestyle interventions that include moderate-intensity activity and dietary modifications are successful in decreasing body weight by more than 4 kg compared to medication and control groups. In addition, those in the lifestyle group had similar, if not better outcomes related to diabetic markers than those in the medication and control group over a four-year period [3]. Thus, we can we can theorize that lifestyle programs may be better suited to prevent the development of type 2 diabetes mellitus (T2DM).

Today, a clear link between obesity and the risk of developing T2DM exists [4,5]. According to the World Health Organization, 39% and 13% of adults over 18 years are considered overweight and obese, respectively [6]. Evidence suggests the prevalence of obesity will rise 33% over the next two-decades [7], while the prevalence of T2DM worldwide will continue to rise from 2.8% in 2000 to 4.4% by 2030 [8]. A recent investigation suggested inadequate physical activity and central obesity are the most common risk factors for T2DM, with age being the greatest predictor of the disease [5]. To date, and contrary to other major global health issues, such as smoking and childhood malnutrition, which have seen decreases in prevalence over time [9,10], obesity interventions have not resulted in significant reductions in worldwide prevalence. As such, it is important to find strategies that will mitigate the development of the disease and encourage individuals to become and remain active.

High intensity functional training (HIFT) uses a variety of exercise modalities, such as mono-structural aerobic activities (e.g., running, rowing), body weight movements (e.g., squats, push-ups), and weightlifting derivatives (e.g., snatch, shoulder press, deadlift) [11]. Unlike high-intensity interval training (HIIT), which tends to focus on singular exercise modalities (e.g., running), HIFT emphasizes functional, multi-joint movements that can be modified to any fitness level, elicit greater muscle recruitment via both aerobic and muscle-strengthening exercises [11,12], and may impact glucose uptake among individuals with T2DM [13,14]. Recently, Feito et al. reported significant reductions in body fat percentage (~6.5%) after 16-weks of training among a group of healthy adults [15]. Additionally, Heinrich et al. showed HIFT was an effective strategy in maintaining adherence and enjoyment among sedentary adults [16]. Thus, it seems HIFT may be a potentially beneficial strategy for combating obesity and potentially the development of T2DM.

Aside from two recent publications from the same study [17,18], which provided insight into the single-group effects of a 6-week HIFT-style intervention on metabolic adaptations, to our knowledge, no other study has compared HIFT training to more traditional exercise programs, incorporating aerobic and resistance training exercises. In the first study, Nieuwoudt et al. [17] showed significant reductions in body fat (−1.1 ± 0.3%, *p* = 0.002) and non-statistical changes in body weight (−1.8 ± 1.0 kg, *p* = 0.09). However, no significant changes were seen for any marker related to glucose control (i.e., glucose, insulin, etc.). Nonetheless, investigators reported improvements in beta-cell function after 6-weeks of training, suggesting that perhaps longer interventions are needed to see statistically significant changes in glucose markers. Meanwhile, Fealy et al. [18] reported significant improvements in insulin sensitivity of ~15%, while reducing total and regional fat mass and maintaining lean mass, which has been shown to be important in the regulation of blood glucose [19]. Therefore, considering the limited number of studies currently available comparing HIFT programs to traditional training programs, the purpose of this study was to examine the differences in body composition (body fat percentage, fat mass, and lean body mass) and glucose control between a combined aerobic and resistance exercise training program (A-RT) and a shorter-duration, high-intensity functional training (HIFT) program in overweight and obese physically inactive adults. Even though we expected both groups to show improvements, we hypothesized that the HIFT group would have greater improvements in body composition and glucose control variables compared to the A-RT group.

## 2. Materials and Methods

### 2.1. Study Design and Participants

This was an eight-week randomized controlled trial conducted between February and May 2012. The consolidated standards of reporting trials guidelines [20] were followed and the study was registered at clinicaltrials.gov (NCT02185872). Inclusion criteria included non-smoking males and females between 18–40 years-old, with a body mass index (BMI) of 25 to <40 kg/m^2^, who were physically inactive (i.e., not participating in any structured exercise programs for the past two months and not exceeding 30 total minutes of physical activity per week), who were not pregnant or planning on getting pregnant during the intervention, who were not taking any blood glucose altering medications, who had no diagnosis of heart disease, or type 1 or T2DM, and had a total cholesterol <200 mg/dL. Participants who had been diagnosed with pre-diabetes were eligible to participate. Participants were not discriminated by age, gender, or ethnicity and similar subjects were randomized to each experimental group through random stratified assignment (age, body mass index ranges).

Participants were recruited via flyers, word of mouth, radio public service announcements, on-campus recruitment, email blast, posters at local businesses, and referrals throughout the University’s surrounding community. Interested participants completed a screening form to determine eligibility, which included questions related to height, weight, age, weekly minutes of physical activity, and general health questions. Individuals meeting the eligibility criteria were then given an informed consent form, a medical history questionnaire, and a demographic information sheet. All study protocols and procedures were approved by the University’s institutional review board (IRB; #6058).

Group randomization was conducted with all participants coded by age and BMI and assigned a number based upon each characteristic. Median and mean values were calculated for age and BMI and cut points were assigned each (i.e., age < or ≥ 28 years; BMI < or ≥ 30.5 kg/m^2^). A random number generator was used to assign participants to each group resulting in 11 participants in the A-RT group (eight females, three males) and 12 (seven males, five females) in the HIFT group. All participants were asked to continue their normal dietary intake and to refrain from doing any extra physical activity outside of the study.

### 2.2. Measures

Participants completed measures the week prior to and the week following the eight-week intervention. Participants were asked to fast eight hours overnight before completing the body composition and anthropometry measures, followed by the fasting plasma glucose measure.

#### 2.2.1. Body Composition and Anthropometry

Body weight and height were measured to the nearest 0.1 kg and 0.1 cm, respectively. Following standard guidelines, a BMI < 18.5 was defined as underweight, 18.5–24.9 was normal weight, 25–29.9 was overweight, and ≥30 was obese [21]. Waist circumference was measured by placing a measuring tape 1” above the umbilicus around the waist of the participant and body composition (e.g., body fat (%), lean body mass (kg), fat mass (kg)) was measured via dual energy X-ray absorptiometry (DXA; Lunar iDXA, GE Healthcare, Madison, WI; USA) scan [22]. Of note, the DEXA body composition measurements were delayed for two weeks at posttest due to a computer malfunction. Participants were neither asked to refrain from participating in any physical activity during the delay with the DEXA, nor was their activity monitored.

#### 2.2.2. Heart Rate

Resting heart rate was recorded using POLAR H7 heart rate monitors (Bethpage, NY, USA) with a chest strap (beats per minute, bpm) after participants were seated for at least three minutes and this was used to calculate heart rate reserve (HRR; Max HR − Resting HR = HRR). HRR was used to determine appropriate exercise intensities for the A-RT program [23].

#### 2.2.3. Glucose Control

Fasting plasma glucose (FPG) levels were taken first thing in the morning via finger stick after an overnight fast of 10–12 h. Participants were specifically asked to refrain from eating or drinking anything other than water prior to the test. Immediately following the FPG test, an oral glucose tolerance test (OGTT) was administered. Participants were given a 75 g oral dextrose solution to consume. Time 0 was documented for each participant at the time of ingestion of the entire solution. Finger sticks were then taken at 30 min, 45 min, and 60 min from time 0.

### 2.3. Exercise Groups

Both exercise groups, A-RT and HIFT, could choose to attend either a morning or evening session on Mondays, Wednesdays, and Fridays.

#### 2.3.1. Aerobic and Resistance Training (A-RT) Exercise Group

The A-RT activities were based upon the Academy of Nutrition and Dietetics and American College of Sports Medicine (ACSM) guidelines to meet the 150 min of moderate-intensity aerobic physical activity plus at least two days of muscle strengthening exercises per week [23]. All A-RT sessions were supervised by an American Council on Exercise (ACE)-certified member of the research team in order to ensure participants were completing the entire protocol accurately, to answer any questions about exercise movements, and to demonstrate proper technique and form when necessary. Participants completed three weekly exercise sessions on Mondays (50 min of aerobic and full-body resistance training), Wednesdays (same as Mondays), and Fridays (50 min of aerobic training). Participants began each training session by checking in and receiving a heart rate strap and heart rate monitor as well as an individualized workout plan. Aerobic activities were completed on machines (i.e., Concept 2 rower, Quinton or Precor treadmill, Precor or Vision Fitness elliptical, or Vision Fitness stationary bike); participants were allowed to switch between machines as long as they had completed at least 10 min towards their 50 total minutes. Intensity was prescribed as 40–50% HRR for weeks 1–4 and 50–60% HRR for weeks 5–8 and participants utilized their heart rate monitors to ensure meeting the intensity prescriptions.

Full-body resistance training exercises took approximately 20 min, with one minute of rest between all sets and exercises. Crunches (3 sets of 15) were completed both days. Participants established one-repetition maximums (1RMs) during week 1 on each CYBEX machine exercise and exercises varied between Mondays (i.e., bicep curls, military presses, lat pulldowns, and leg extensions) and Wednesdays (i.e., tricep pulldowns, bench presses, reverse leg curls, and seated leg presses). Participants performed three sets of each exercise using the following progressive repetition scheme: Weeks 2–3: 15 reps at 50% 1RM; Weeks 4–5: 12 reps at 60% 1RM; Weeks 6–7: 10 reps at 70% 1RM; Week 8: 8 reps at 75% 1RM).

#### 2.3.2. High Intensity Functional Training (HIFT) Exercise Group

A total of 24 sessions lasting up to 60 min were included in the HIFT program. CrossFit was the HIFT program used in this study. All HIFT sessions were led by a CrossFit Level 2 trainer and the first two sessions were structured as an introduction to common movements used in HIFT (e.g., squats, deadlift, press, jerks, barbell, dumbbell, and medicine ball cleans, pull-ups, kettlebell swings, among others); no additional workouts were completed on days 1 and 2. Beginning on day 3, each HIFT class consisted of 10–15 min of stretching and warmup, 10–20 min of instruction and practicing techniques and movements, and 5–30 min for the workout of the day (WOD), performed at vigorous intensity, relative to each person’s ability and fitness level. Workout modality components included aerobic (e.g., running, jumping rope), body weight (e.g., pull-ups, squats), and weightlifting (e.g., front squats, kettlebell swings) exercises that were constantly varied using the CrossFit training template [24] in single, couplet, or triplet modalities that were completed for time, repetitions, or weight. All weights and movements were individually prescribed and recorded for each HIFT participant. Example workouts from Week 4 of the study have been previously published [16]. Depending on the WOD structure, the times to complete the WOD, rounds and repetitions completed on the WOD, weights used, and any modifications needed from the programmed workout were also recorded for each participant. Average times for each WOD and total average WOD time per week were calculated for the HIFT group as a whole.

### 2.4. Incentive

After the conclusion of the intervention, participants were provided with one free month of membership to the university department’s adult fitness program or CrossFit classes.

### 2.5. Statistical Analysis

All data were entered into IBM-SPSS 25 (Armonk, NY, USA) for statistical analyses and comparisons. Due to the pilot nature of this study, an a priori sample size calculation was not computed. Descriptive statistics including means (M), standard deviations (SD), and percentages (%) were computed for all participant demographics by group. We used the equation [(Time 45 – Time 30) × 1⁄2 (OGTT 30 + OGTT 45)] + [(Time 60 – Time 45) × 1⁄2 (OGTT 60 + OGTT 45)] to calculate glucose area under the curve (AUC) using the trapezoidal method [25]. Independent samples *t*-tests were conducted on each measure to determine differences between groups at baseline. Paired-sample t-tests were conducted to examine within-group changes in all variables of interest (e.g., glucose AUC) from pretest to posttest. Additionally, a 2 (group) × 2 (time) repeated measures analysis of variance (ANOVA) was conducted to evaluate between-group differences for changes from pretest to posttest. Effect sizes (Cohen’s *d)* were calculated to examine further differences between the groups. Effects sizes were considered small (*d* = 0.20), medium (*d* = 0.50), and large (*d* = 0.80) based on published standards [26]. All data are presented as M ± SD unless otherwise noted. For all analyses, the alpha level was set at 0.05.

## 3. Results

### 3.1. Participant Characteristics and Baseline Measurement Values

Eighteen participants completed the intervention (nine in the A-RT group: seven females, two males; and nine in the HIFT group: five females, four males) by attending or making up at least 90% of the 24 scheduled exercise sessions on alternate days. Only one participant reported having pre-diabetes, but he did not complete the intervention. The study attrition rate was 21.7% (A-RT = 18.2% vs. HIFT = 25%). Two male participants, one from each group, cited scheduling issues as their reason for dropping out of the study and one male from the HIFT group stated a lower body injury and groin muscle pull during an exercise session as his reason for discontinuing the intervention. The other two participants (an A-RT female and a HIFT male) did not provide a reason for discontinuing their participation.

Groups were similar in age (AR-T: 25.9 ± 4.2 years vs. HIFT: 27.7 ± 6.7 years). There were more females in the AR-T group (77.8%) compared to the HIFT group (55.6%) and the HIFT group had a larger proportion of individuals who reported being employed (AR-T: 55.6% vs. HIFT: 77.8%), as well as reported being married (AR-T: 33.3% vs. HIFT: 55.6%). Both groups had similar proportion of individuals with a bachelor’s degree or above (AR-T: 77.8% vs. HIFT: 58.3%). No significant differences in baseline measurements were found between groups (see Table 1).

### 3.2. Time Spent Exercising 

Total training session times were 190 min per week for the A-RT group and 180 min for the HIFT group. As previously reported [16], the A-RT group spent significantly more time on average completing workouts each day (A-RT M = 63.3 ± 3.3 min vs. HIFT M = 13.3 ± 6.4 min, *t*(16) = 43.5, *p* < 0.001) and each week (A-RT M = 189.8 ± 10.0 min vs. HIFT M = 39.3 ± 2.5 min; *t*(16) = 43.7, *p* < 0.001).

### 3.3. Between-Group Differences for Body Composition and Glucose Variables

Within- and between-group differences for key variables are shown in Table 2. Overall, no significant changes were observed for any of the measured body composition variables. Of the body composition variables recorded, only body fat % within HIFT group participants showed a trend towards significance (*t* = −2.04; *p* = 0.08). In addition, neither of the two measures related to glucose control demonstrated significant changes after 8-weeks of training in either of the two groups. Of note, the mean difference for glucose AUC increased for the A-RT group compared to the HIFT group (279.4 ± 404.6 vs. −23.8 ± 577.1, respectively), although there were no significant differences between groups. This change in glucose AUC did trend toward significance for the A-RT group (*t* = 1.95, *p* = 0.09).

Further analysis of regional body composition changes identified significance changes in legs lean body mass within the HIFT group (*t* = 3.3, *p* = 0.01). As shown in Table 3, no other significant within-groups or between-groups differences were found.

## 4. Discussion

The purpose of this study was to examine the impact of standard aerobic and resistance exercise training program (A-RT) and a high-intensity functional training (HIFT) program in overweight and obese, physically inactive adults. Even though we expected both groups to show improvements, we hypothesized that the HIFT group would have greater improvements in body composition and glucose control variables compared to the A-RT group. The only significant difference we found was an increase in legs lean body mass within the HIFT group. While our overall findings did not support our hypotheses, we believe they are noteworthy and helpful for future studies.

Recently, Nieuwoudt et al. [17] investigated changes in beta-cell function after six weeks of HIFT among 12 sedentary adults with documented T2DM. After completing 10–20-min sessions 3 days/week, participants saw significant improvements in beta-cell function, while decreasing body fat and preserving lean mass [17]. In a second paper, Fealy, Nieuwoudt, et al. [18] evaluated the effectiveness of their 6-week HIFT intervention for cardiometabolic risk factors, and reported improvements in blood pressure, body composition, fat oxidation, plasma triglycerides, and very-low density lipoprotein. Additionally, insulin sensitivity was increased after training, although the downward shift in glucose was not statistically significant post-intervention. These two papers provided initial insight for HIFT benefits among individuals with T2DM and supported the notion this type of training was beneficial, even though the total volume of training was lower than typically prescribed [23,27]. To our knowledge, no additional studies have investigated the metabolic changes associated with HIFT. Similarly, studies comparing more typical exercise (e.g., aerobic and resistance training) programs and HIFT programs are also lacking. Thus, our study design is timely and necessary. Of interest, lean body mass did improve in the HIFT group, which shows promise for those with type 2 diabetes. Olsen et al. [28] found blunting of glucose uptake in the legs, which should improve with increased lean body mass. Although we did not find any significant between-group differences, our study provides the first look at the comparison between HIFT and traditional training in glucose control and body composition markers, which has not yet been described in the literature.

Even though our overall study hypotheses were not supported, our findings are not unfounded. Several investigators have supported the notion 8-12 weeks of training may not be enough time to significantly improve glucose metabolism after endurance and resistance training programs for non-diabetic individuals [29,30]. For example, Craig et al. [29] reported on the effect of a 12-week resistance training program among a group of young (23 ± 1 year) and older (63 ± 1 year) male participants. Even though all participants demonstrated improvements in lean mass and body composition, neither group showed changes in glucose response despite lowered insulin response after a glucose load [29]. Similarly, Fenicchia et al. [30] examined the effects of an acute resistance training session, as well as a six-week training program among T2DM women and showed that even though resistance training was effective in increasing strength, glucose concentration improved immediately after the acute bout, but not after six weeks of training. Additionally, insulin concentrations did not seem to improve [30].

Even though several investigators have postulated a potential “exercise resistance”, where a genetic predisposition exists to lower physiological improvements as a result of exercise training [31,32,33], we believe our findings might be related to several potential limitations of our research design. First, due to the pilot nature of our investigator our study sample was small, and may not be generalizable to overweight/obese individuals at large. Several investigators have reported significant improvements in body composition parameters using this type of training [11,34,35,36]. Second, a standardized physical activity questionnaire was not used for the screening process, which may have resulted in participants under-reporting their physical activity levels at time of study enrollment. Third, our groups had an uneven sex distribution which may have affected our results. Fourth, we did not control nor measure dietary intake throughout the duration of the intervention including prior to study measurements, and simply asked participants to continue their normal dietary intake, which may have impacted our results. Even though this type of training promotes low carbohydrate diets, such as Paleo^®^ and Zone^®^ [37] we are uncertain if any our participants started to follow any of these dietary plans during our study. Recent evidence suggests these dietary approaches could be beneficial for weight loss, although data are less clear for glucose control [38]. However, in a 2015 review, Balk et al. suggested that more intensive interventions, using more aggressive exercise prescription including higher intensity, and greater volumes, which could be representative of any HIFT program, are more effective in promoting weight loss and reducing diabetic risk [39]. Fifth, we also did not record information on typical day-to-day error of the DEXA machine. Lastly, it may be that our intervention did not provide significant stimulus for energy deficit, as suggested by current guidelines [21]. Future studies should also be designed to account for dietary intake, where kilocalorie levels are adjusted for the individual’s body weight and physical activity levels [21] and energy expenditure for −500 kcal/d or −750 kcal/d or 30% total energy deficit [21], in order to ensure significant changes in body composition variables. This focused approach is sure to elicit improvements in glucose control, as evidence suggest expending a minimum of 400 kcal/week improves insulin action in sedentary adults [40].

In summary, it is noteworthy and encouraging that this 8-week intervention resulted in relatively low attrition rates (~22%) and participants achieved over 90% adherence of all training sessions. Even though attrition rates for intervention studies can be difficult to quantify [41], most studies that achieve low attrition rates, include additional components such as behavioral training (e.g., goal setting, mastery; [42]), weekly behavioral sessions and exercise logs [43], bi-weekly phone calls [44] and exercise self-monitoring in the form of detailed daily logs [45], which tend to be cumbersome for the participant and impractical in a “real world” scenario. Recently, several investigations from our laboratory have elucidated on the motivational factors that affect HIFT participation [46,47,48] and suggest this type of group-based training that includes constant personal challenges and competition seem to be motivating for most adults. Further studies should be conducted with larger samples to further investigate the effects of HIFT on body composition and glucose control for overweight and obese adults.

## Figures and Tables

**Table 1:** Comparison of baseline body composition and fasting glucose for both study groups.

Variable	A-RT ^1^ M ± SD	HIFT ^2^ M ± SD	*t*	*p*-Value	Cohen’s *d*
Weight (kg)	86.1 ± 9.7	86.6 ± 10.1	−0.11	0.91	0.05
Body Mass Index (kg/m^2^)	30.1 ± 3.5	30.9 ± 2.3	−0.52	0.61	0.27
Waist Circumference (cm)	91.8 ± 8.8	97.2 ± 7.0	−1.45	0.17	0.68
Body Fat (%)	43.7 ± 7.2	40.5 ± 7.4	1.04	0.31	0.44
Fat Mass (kg)	35.6 ± 7.1	33.2 ± 6.7	0.74	0.47	0.35
Lean Body Mass (kg)	46.5 ± 9.3	49.0 ± 10.4	−0.54	0.60	0.25
Fasting Plasma Glucose (mg/dL)	87.3 ± 8.1	88.4 ± 10.6	0.29	0.78	0.12
Glucose AUC	4292 ± 677	4448 ± 793	0.49	0.63	0.21

^1^ A-RT. Aerobic and Resistance Training (n = 9). ^2^ HIFT. High Intensity Functional Training (n = 9).

**Table 2:** Within- and between-group differences in body composition and glucose variables at posttest.

Variable	A-RT ^1^ M Difference ± SD	HIFT ^2^ M Difference ± SD	Cohen’s *d*	ƒ	*p*-Value
Weight (kg)	−0.4 ± 1.4	−0.4 ± 1.6	0.00	0.00	0.97
Body Mass Index (kg/m^2^)	−0.1 ± 0.6	−0.2 ± 0.7	0.15	0.24	0.63
Waist Circumference (cm)	−2.4 ± 4.8	−1.4 ± 5.1	0.20	0.18	0.68
Body Fat (%)	−0.3 ± 1.7	−1.1 ± 1.6	0.48	0.94	0.35
Fat Mass (kg)	−0.6 ± 1.5	−1.0 ± 1.7	0.25	0.34	0.57
Lean Body Mass (kg)	0.1 ± 1.6	0.6 ± 1.2	0.35	0.57	0.46
Fasting Plasma Glucose (mg/dL)	1.4 ± 8.4	0.4 ± 14.0	0.09	0.03	0.86
Glucose AUC	279.4 ± 404.6 ^3^	−23.8 ± 577.1	0.61	1.58	0.23

^1^ A-RT. Aerobic and Resistance Training (n = 9). ^2^ HIFT. High Intensity Functional Training (n = 9). ^3^ n = 8. Note that all within-groups paired samples t-tests had *p*-values >0.05.

**Table 3:** Within- and between-group differences in regional body composition at posttest.

Variable	A-RT ^1^ M Difference ± SD	HIFT ^2^ M Difference ± SD	Cohen’s *d*	ƒ	*p*-Value
Arms Lean Body Mass (kg)	0.02 ± 0.33	0.17 ± 0.65	0.29	0.35	0.56
Arms Fat Mass (kg)	−0.03 ± 0.21	−0.10 ± 0.35	0.24	0.24	0.63
Trunk Lean Body Mass (kg)	−0.14 ± 1.20	−0.22 ± 1.53	0.06	0.02	0.90
Trunk Fat Mass (kg)	0.02 ± 0.87	−0.90 ± 1.57	0.69	2.49	0.13
Legs Lean Body Mass (kg)	0.13 ± 0.84	0.53 ± 0.48 ^3^	0.58	1.53	0.23
Legs Fat Mass (kg)	−0.41 ± 0.82	−0.03 ± 0.58	0.54	1.28	0.27

^1^ A-RT. Aerobic and Resistance Training (n = 9). ^2^ HIFT. High Intensity Functional Training (n = 9). ^3^ *p* = 0.01 for within-group differences.
